# Mid-term results of arthroscopic Bankart repair and remplissage for recurrent anterior shoulder instability in patients with a history of seizures

**DOI:** 10.1186/s12891-021-04960-9

**Published:** 2022-01-03

**Authors:** Mohammad Reza Guity, Amir Sobhani Eraghi

**Affiliations:** 1 Orthopaedics Department, Imam Khomeini Hospital, Tehran University of Medical Sciences, Tehran, Iran; 2 Orthopaedics Department, Rasool Akram Medical Complex, Iran University of Medical Sciences, Tehran, Iran

**Keywords:** Shoulder dislocation, Epilepsy, Arthroscopy

## Abstract

**Background:**

Seizure predisposes patients to shoulder dislocation. However, there is no consensus regarding the best management approach for recurrent shoulder dislocation in patients who have a history of seizures. In this study, we report the outcome of arthroscopic Bankart repair augmented by Remplissage for the recurrent anterior shoulder dislocation in a series of patients with a history of seizures.

**Methods:**

In this retrospective study, 27 patients with 29 recurrent anterior shoulder dislocations who were treated with the arthroscopic Bankart repair were included. All cases had deep Hill-Sachs lesions according to Hardy classification that was managed with a Remplissage technique. Patients with a glenoid defect of more than 20% in the CT scan were excluded. Twenty-two patients had an epileptic seizure, while the remaining five patients had convulsions due to other causes. The mean age of the patients was 28.3 ± 6.2 years. The mean follow-up of the patients was 3.1 ± 1.2 years. Outcome measures included the shoulder range of motion that was compared with the non-injured side in the unilateral subjects and the shoulder function that was evaluated by the Rowe score and the Walch-Duplay score.

**Results:**

The mean forward flexion, abduction, external rotation, and internal rotation were not significantly different between injured and non-injured shoulder (*p* = 0.34, *p* = 0.41, *p* = 0.11, *p* = 0.23). The mean Rowe score was 49.1 ± 7.8 before the surgery and 92.1 ± 6.4 at the last visit (*p* < 0.001). According to the Walch-Duplay score, the shoulders were categorized as excellent, good, and fair in 17 (58.7%), 11 (37.9%), and 1 (3.4%) shoulder, respectively. The overall rate of instability recurrence was 17.2% (*n* = 5).

**Conclusion:**

In patients with a history of seizures, arthroscopic Bankart repair augmented by Remplissage could be regarded as a safe and efficient method for the treatment of recurrent anterior shoulder dislocation with glenoid defect < 20%.

## Introduction

The shoulder joints are considered the most commonly dislocated joint in the human body, comprising up to 50% of all human joint dislocations [1]. The rate of re-dislocation is high following the initial dislocation, which restricts the patients’ daily activities [2]. Arthroscopic treatment of shoulder dislocation is a minimally invasive approach that has significantly evolved over the last decades and offers encouraging results with several advantages over the open surgical treatment, including a decreased rate of morbidity, early functional rehabilitation, improved range of motion, less hospital stay, and lower costs [3].

Nowadays, the most common surgical procedure for the treatment of recurrent anterior shoulder instability is arthroscopic Bankart repair, but some factors such as young age, sportive activities, and glenoid defects may result in a high failure rate, and patients with these characteristics could benefit from open surgery or arthroscopic anterior bone block procedures. In addition to the Bankart repair, addressing Hill-Sachs defect (the so-called Remplissage technique) had lowered the failure rate of arthroscopic surgery without any significant restriction of range of motion [4].

Patients who have a seizure attack are susceptible to shoulder dislocation [5]. By contrast to the general population, a posterior shoulder dislocation is more frequent than anterior dislocation in epileptic patients [5, 6]. Shoulder dislocation in the context of seizures is highly prone to recurrence. This is due to the severity of initial pathologies, especially huge defects of the humeral head and glenoid. The recurrence of convulsion attacks aggravates the problem [5, 7, 8]. Despite its importance, treatment of shoulder dislocation in epileptic conditions has only been discussed in a small number of studies [7, 9].

To the best of our knowledge, no study has been performed to evaluate the outcome of arthroscopic Bankart repair augmented by Remplissage for the treatment of recurrent anterior shoulder dislocation in epilepsy or other convulsive disorder. Considering the advantages of this technique, we hypothesized that it could provide satisfactory outcomes for the treatment of seizures-associated anterior shoulder dislocation, thereby encouraging the surgeons to more comprehensively implicate this approach. In this study, we tested this hypothesis.

## Patients and methods

### Study design

This retrospective study was approved by the review board of our institute, and patients provided written consent before participation in the study. Between 2014 and 2018, 48 patients with a recurrent anterior shoulder dislocation and a history of seizures who were referred to our university hospital were evaluated for eligibility criteria. The inclusion criteria were the age of > 16 years, first dislocation occurring during seizures, history of at least two anterior shoulder dislocations due to seizures, and well-controlled epilepsy under the supervision of a neurologist so that the patient was seizure-free for at least 1 year. Patients with follow-up of fewer than 2 years (*n* = 2), a history of shoulder stabilizing surgery (*n* = 1), uncontrolled seizures (*n* = 3), severe mental retardation (n = 1), severe hyperlaxity with multidirectional or voluntary instability (*n* = 2), and patients with a glenoid defect of more than 20% in the CT scan (*n* = 12) were excluded from the study. Finally, 29 anterior shoulder dislocations from 27 patients were included in the study. The study population included 23 (85.2%) males and four (14.8%) females with a mean age of 28.3 ± 6.2 years (range 17–42). The mean follow-up of the patients was 3.1 ± 1.2 years (range 2–5). The mean interval between the last seizures and the surgery was 25.4 ± 3.8 months (range 18–32). The etiology of seizures included generalized seizures or Tonic-Clonic seizures (*n* = 22), insulin-dependent diabetes mellitus who had hypoglycemic convulsion during sleep (*n* = 3), and tramadol abuse (n = 2). Six patients reported shoulder pain after the first episode of convulsion, but their shoulders dislocated spontaneously after a while. The mean number of episodes of dislocations was 8.6 ± 2.2 (range 2–12). The mean time interval between the first dislocations to surgery was 30.2 ± 5.3 months (range 22–36). The characteristic features of the patients are demonstrated in Table 1 in detail.

**Table 1:** Characteristic features of patients with recurrent anterior shoulder dislocation attributed to the seizure

Variable	Epileptic patients (***n*** = 27)
**Age (year)**	28.3 ± 6.2
**Gender**	
**Male**	23 (85.2)
**Female**	4 (14.8)
**Follow-up (year)**	6.7 ± 3.2
**Time interval from the first dislocation to surgery (month)**	30.2 ± 5.3
**Time interval from final seizure to surgery (month)**	25.4 ± 3.8
**Mean number of dislocation**	8.6 ± 2.2
**Etiology of Seizure**	
**Generalized seizure**	22 (81.5)
**Diabetic hypoglycemia**	3 (11.1)
**Tramadol consumption**	2 (7.4)
**Laterality**	
**Right**	21 (77.7)
**Left**	4 (14.8)
**Bilateral**	2 (7.5)

Data are presented as mean ± SD or number & percentage

A three-dimensional CT scan was obtained for all patients to evaluate possible anterior glenoid erosion and to measure the depth of Hill-Sachs lesion according to Hardy classification [10]. Accordingly, the mean glenoid erosion was 12.1 ± 8.9% (range 0–18). The mean Hardy index was 24.3 ± 6.8 (range 14–31). Bony Bankart was evident on CT scan in 14 cases and was not regarded as a contraindication for arthroscopic surgery.

### Surgical procedure

All the surgeries were performed by one fellowship-trained shoulder surgeon. Under general anesthesia, the patient was placed in a Beach chair position. The standard posterior, anterosuperior, and anteroinferior portals were established. A 7 mm cannula was placed in the anterosuperior portal and an 8.5 mm cannula into the anteroinferior portal. Since in all of our cases significant Hill-Sachs lesion was found on preoperative CT scan, the Remplissage technique was considered as an integrated part of the surgery, and we did not rely on intraoperative Hill-Sachs lesion engagement for doing this procedure. A small 5 mm cannula was inserted in the posterolateral (Wilmington) portal for the Remplissage. After complete diagnostic arthroscopy through posterior and anterosuperior portals, the first part of the Remplissage procedure was begun by light decortication of the Hill–Sachs lesion. Two 2.8 mm titanium anchors (Arthrex) were then introduced via the posterolateral portal, one in the most medial and the other one in the most lateral part of the humeral head defect; both anchors were placed just lateral to the cartilage. Then, using a periosteal elevator, the capsulolabral complex was mobilized from the anterior surface of the glenoid. The neck of the glenoid was slightly abraded with an arthroscopic burr. By using three knotless anchors (2.5 mm PUSHLOCK, ARTHREX) placed at 6, 4, and 2 o’clock, the capsulolabral complex was repaired back to the glenoid rim. In the end, the scope was moved into the subacromial space, and by using the double pulley technique, the sutures of anchors placed in the Hill–Sachs defect were tied together to complete the Remplissage procedure. After the surgery, the patients’ shoulder was immobilized in a sling for 6 weeks. Pendular motion exercises were ordered for the first 2 weeks, followed by elevating the elbow to shoulder level from the fourth week. During the next 2 months, the range of motion progressed to full movements with physiotherapy. Follow-up visits were performed regularly according to our institution’s protocol.

### Outcome measures

The included patients were called and asked to attend a final evaluation session. At the evaluation session, the shoulder stability was assessed clinically with an overhead maneuver and apprehension test. Range of motion (ROM) was compared between injured and non-injured shoulder in unilateral injuries and included forward elevation, abduction, external rotation, and internal rotation. Subjective measures of the outcome included the evaluation of the shoulder strength and functional indexes using the Rowe score [11] and the Walch-Duplay score [12]. The Rawe score was evaluated before and after the surgery. The Walch-Duplay score was evaluated at the last follow-up. The Rowe scores were categorized as Excellent (90–100), good (75–89), average (51–74), and bad (< 54). The Walch-Duplay scores were categorized as excellent (91–100), good (76–90), fair (51–75), and poor (< 50). Postoperative complications were extracted from the patients’ medical profiles.

### Statistical analysis

SPSS for Windows version 16 (Chicago, Illinois, USA) was used for the statistical analysis. Descriptive data were presented as mean ± standard deviation or number & percentage. A paired t-test was used for the comparison of preoperative and postoperative Rowe scores. An independent T-test or its nonparametric counterpart (Mann-Whitney U test) was used for the comparison of the mean difference of shoulder movements between the injured and healthy sides. The Kruskal-Wallis test was used for the comparison of ordinal variables. Pearson’s or Spearman’s correlation coefficient test was used for the assessment of potential correlations. A *P*-value of < 0.05 was regarded as statistically significant.

## Results

The outcome of the arthroscopic Bankart repair augmented by Remplissage was evaluated in 27 patients with 29 anterior shoulder dislocations. The mean difference of the forward flexion between injured and non-injured shoulders was 1.3 ± 4.2° (*p* = 0.34). The mean difference of the shoulder abduction between the injured and non-injured sides was 1.2 ± 4.6° (*p* = 0.41). The mean difference of the external and internal rotation between the injured and contralateral shoulder was 3.8 ± 4.2° and 1 ± 1.8°, respectively (*p* = 0.11, and *p* = 0.23, respectively). The detailed shoulder ROM is demonstrated in Table 2.

**Table 2:** Comparison of the range of motion between operated and non-operated shoulders (bilateral cases were excluded)

Motion	Operated shoulder (***n*** = 25)	Non-operated shoulder (***n*** = 25)	***P***-value
**Forward elevation (°)**	171 ± 8.6	172.3 ± 7.9	0.34
**Abduction (°)**	170 ± 7.1	171.2 ± 7.6	0.41
**External rotation (°)**	58.6 ± 17.2	62.4 ± 18.3	0.11
**Internal rotation (°)**	64.7 ± 11.2	65.7 ± 13.9	0.23

The mean Rowe score was 49.1 ± 7.8 before the surgery and 92.1 ± 6.4 at the last follow-up (*p* < 0.001). Before the surgery, the Rowe scores were bad in 21 (72.4%) shoulders and average in 8 (27.6%) shoulders. After the surgery, the Rowe scores were excellent in 22 (75.9) shoulders, good in 5 (17.3) shoulder, average in 1 (3.4%) shoulder, and bad in 1 (3.4%) shoulder (*p* < 0.001).

At the final evaluation, the mean Walch-Duplay score was 89.6 ± 11.3. According to the Walch-Duplay scores, the shoulder function and strength was excellent, good, and fair in 17 (58.7%), 11 (37.9%), and 1 (3.4%) patient, respectively. Poor Walch-Duplay score was not observed in any of the dislocated shoulders of this series.

No significant difference was observed between the mean postoperative Rowe score and the Walch-Duplay score of male and female patients (*p* = 0.68, and *p* = 0.71, respectively). The time interval from last seizures to surgery was not correlated with the ROWE score (r = 0.089, *p* = 0.59) and the Walch-Duplay score of the patients (r = 0.077, p = 0.68).

During the follow-up period, seven patients experienced new seizures. The shoulder dislocation recurred in five of these patients. The re-dislocation rate was 17.2% (5 of 29). Four of these patients were managed with the Latarjet procedure. The other patient declined further treatment. Two other patients with new seizures episodes had an apprehension sensation without overt dislocation.

## Discussion

The most important finding of this study was the satisfactory results of arthroscopic Bankart repair augmented by Remplissage in the treatment of seizures-associated anterior shoulder dislocation. The mean shoulder ROM was comparable between the affected and non-affected sides. The Rowe score significantly was improved at the last follow-up visit. The Walch-Duplay score was in the acceptable range in all patients so that no poor outcome was observed in any of the subjects. Re-dislocation occurred in five (17.2%) patients and was attributed to postoperative seizures.

Raiss et al. evaluated the outcome of the Latarjet procedure in the treatment of anterior shoulder dislocation in 14 shoulders from 12 epileptic patients. A bony defect was present in the glenoid rim of all shoulders. At a mean follow-up of 8.3 years, mean shoulder forward flexion and external rotation revealed significant improvement. The mean Rowe score was 76. Re-dislocation occurred in six patients (43%) during a seizure. Due to the high rate of re-dislocation, they suggested Latarjet procedure as an option only for the treatment of well-controlled epileptic patients [9]. The mean Rowe score of the patients in the present series was 92.1, which was significantly higher than the mean Rowe score of the study of Raiss et al. Re-dislocation rate was significantly lower in our patients in comparison with the study of Raiss et al. (17.2% vs. 43%). However, it should be noted that the percentage of patients who experienced postoperative seizures was significantly more in the study of Raiss et al. (83.3% vs. 55.1%). In addition, we only included patients with well-controlled seizures and glenoid defects of < 20%.

Buhler et al. evaluated the outcome of 34 shoulder dislocations in 26 patients in whom the initial dislocation was caused by epileptic seizures. The dislocation was anterior in 17 shoulders. Large bony defects were present in 10 out of 17 shoulders. All the patients were treated by open surgery and different procedures. At a mean follow-up of 10 years, the re-dislocation rate was 47% (8/17). The final result was good in 13 cases, satisfactory in 2 cases, and unsatisfactory in 2 cases. Twelve out of 13 shoulders that were treated with skeletal reconstruction were stable. They concluded that skeletal reconstruction is necessary to obtain clinical stability [7]. The rate of re-dislocation was significantly lower in the present series.

Hutchinson et al. devised a bone buttress operation to secure the deficient anterior glenoid using a bone graft in patients with anterior shoulder dislocation. Fourteen epileptic patients with anterior shoulder dislocation were included in this study. After the surgery, no re-dislocation was observed in patients. Despite a small restriction in the shoulder range of movement, all patients were satisfied with the result. They suggested this surgical approach as the approach of choice for the treatment of problematic cases [6]. Rationally, for patients with a small bony defect, a less invasive technique such as arthroscopy could be regarded as a more judicious approach. The results of the present series approve such a deduction.

Thangarajah et al. retrospectively reviewed the outcome of shoulder dislocation in 33 epileptic patients with 49 unstable shoulders. Thirty-six shoulders were dislocated anteriorly. Large Hill-Sachs lesions and significant glenoid bone loss were present on 21 and 11 shoulders, respectively. Thirty-six shoulders from 31 patients were treated by open surgery. Postoperative instability occurred in 61% (22 of 36 shoulders) of shoulders with anterior dislocation. Skeletal reconstruction was associated with a significantly lower rate of re-dislocation. Glenohumeral arthrosis was detected in 22 (61.1%) shoulders at a mean follow-up of 12 years. They concluded that the presence of bone loss is a determining factor in the selection of surgical strategies in epileptic patients [13]. Based on our results, the arthroscopic Bankart repair augmented by Remplissage could be an acceptable surgical choice for the management of recurrent anterior shoulder dislocation in patients with a history of seizures who do not have significant glenoid bone loss.

The potential clinical relevance of the present study could be using the arthroscopic Bankart repair augmented by Remplissage for the treatment of recurrent anterior shoulder dislocation in epileptic patients with a well-controlled status of seizures episodes. We believe that good control of the patients’ seizures reduces the rate of re-dislocation, thereby improving the outcome of treatment. Therefore, we suggest delaying the surgery until the patient’s seizures is well controlled.

The strict inclusion and exclusion criteria could be regarded as the main strength of this study, thereby increasing the likelihood of more reliable and reproducible results through reducing the heterogeneity of study populations.

The main limitation of this study was its retrospective design. The other limitation of the study was the relatively short period of follow-up, as spontaneous recurrence and further seizures may occur later in patients’ life, compromising the overall good results.

## Conclusion

In selected epileptic patients and other convulsive disorders presented with a recurrent anterior shoulder dislocation and glenoid defect < 20%, arthroscopic Bankart repair augmented by Remplissage provides favorable functional outcomes with relatively small rate of re-dislocation. Therefore, this procedure could be suggested as a safe and efficacious method in the treatment of this group of patients.

## Data Availability

The authors agree with sharing, coping, and modifying the data used in this article, even for commercial purposes. The datasets used and analysed during the current study are available from the corresponding author on reasonable request.

## Abbreviation

ROM: Range of motion
